# Design and Implementation of a Position, Speed and Orientation Fuzzy Controller Using a Motion Capture System to Operate a Wheelchair Prototype

**DOI:** 10.3390/s21134344

**Published:** 2021-06-25

**Authors:** Mauro Callejas-Cuervo, Aura Ximena González-Cely, Teodiano Bastos-Filho

**Affiliations:** 1 Software Research Group, Universidad Pedagógica y Tecnológica de Colombia, Av. Central del Norte 39-115, Tunja 150001, Colombia; aura.gonzalez@uptc.edu.co; 2 Posgraduate Program in Electrical Engineering, Federal University of Espírito Santo, Av. Fernando Ferrari, 514, Vitoria 29075-910, Brazil; teodiano.bastos@ufes.br

**Keywords:** inertial measurement unit, fuzzy control, motion capture, position, velocity, orientation, head movements, inertial sensor

## Abstract

The design and implementation of an electronic system that involves head movements to operate a prototype that can simulate future movements of a wheelchair was developed here. The controller design collects head-movements data through a MEMS sensor-based motion capture system. The research was divided into four stages: First, the instrumentation of the system using hardware and software; second, the mathematical modeling using the theory of dynamic systems; third, the automatic control of position, speed, and orientation with constant and variable speed; finally, system verification using both an electronic controller test protocol and user experience. The system involved a graphical interface for the user to interact with it by executing all the controllers in real time. Through the System Usability Scale (SUS), a score of 78 out of 100 points was obtained from the qualification of 10 users who validated the system, giving a connotation of “very good”. Users accepted the system with the recommendation to improve safety by using laser sensors instead of ultrasonic range modules to enhance obstacle detection.

## 1. Introduction

This research concerns the design and implementation of a position, speed, and orientation controller in a wheelchair simulation prototype through the use of graphical interface, inertial systems, and digital control algorithms. To introduce the topic, according to the World Health Organization (WHO), “Disability is the umbrella term for impairments, activity limitations and participation restrictions, referring to the negative aspects of the interaction between an individual (with a health condition) and that individual’s contextual factors (environmental and personal factors)” [1]. The causes of the functional performance impairments of the human body are linked to problems in pregnancy, growth, spinal cord injuries, and organ dysfunctions. Physical disabilities are associated with sedentary behavior [2], cerebral palsy, diplegia, dislocations, contractures, scoliosis [3], and spina bifida [4]. They also involve neural tube defects related to birth issues in the brain, vertebral column and/or spinal cord [5]. Therefore, the proposal of viable solutions to such impairments are desirable, allowing the use of assistance mechanisms that improve patient mobility.

According to the latest fact sheets from WHO, “over 1 billion people are estimated to live with some form of disability. This corresponds to about 15% of the world’s population, with up to 190 million (3.8%) people aged 15 years and older having significant difficulties in functioning, often requiring healthcare services. The number of people living with disability is increasing, in part due to ageing populations and an increase in chronic health conditions” [6].

Wheelchairs are considered as an assistance instrument and allow the mobility of a person with functional performance disability. They are classified into manual and automatic. The design and construction of a controller for an automatic wheelchair have been studied using different closed-loop control methods [7,8,9,10,11]. These designs are one of the major configurations of control systems based on instrumentation, analog, and/or digital electronics.

Automatic wheelchair digital controllers depend on the type of instrumentation used. There are non-invasive instrumentation systems placed on the user, such as those based on cerebral activity, like Brain Computer Interfaces (BCIs) [12,13,14], those based on inertial and magnetic sensors that measure head or hand movements [15,16,17], and those that implement Electrooculography (EOG) as well as Electromyography (EMG) [18,19], [20]. On the other hand, there are controller systems placed on the wheelchair like those that involve the use of distance sensors to detect obstacles or operate the wheelchair in closed environments [21,22,23], besides those that use vision artificial techniques [24,25,26]. There are other types of instrumentation that depend on the user characteristics, the wheelchair navigation, or the environment, being outdoors or indoors.

The structure of the article is made up of four stages: First, the wheelchair prototype instrumentation and the configuration of the IMOCAP-GIS motion capture system located on the user’s head; second, the mathematical model considering the dynamic systems model theory; third, manual and automatic control by means of intelligent control techniques to operate the prototype in seven directions: Forward, backward, right, left, back-right, back-left, and stop; fourth, a safety control using distance sensors to detect static obstacles. Finally, the evaluation of the performance through a laboratory test protocol that validates the system and user experience.

This work is part of an ongoing investigation. It began with a systematic review developed in [27], in addition to the development of the proposed system in a wheelchair prototype, the upcoming implementation in an electric-powered wheelchair, and experimental tests with users with disabilities in lower and upper limbs.

## 2. Background and Related Works

Wheelchair control systems use different types of instrumentation and control techniques, including head motion controllers that operate a prototype or wheelchair, as indicated by the state of the art that was carried out in [27]. Designs and implementations of controllers were also defined based on the research development of different universities as mentioned by [28]. Among instrumentation types mentioned in the literature review, an important topic is the use of inertial-magnetic sensors to detect body movements, specifically head movements. This type of control does not involve upper limbs movements, thus people with upper/lower limbs disabilities can use this system.

Ruzaij et al. [29] developed a system to operate the wheelchair through an intelligent application with two operation modes: Voice commands and head movements. The sensors used were microelectromechanical systems (MEMS). The design has a compensation speed system in case of going through ascending or descending a ramp, and it depends on the user tilt angle [30]. A calibration algorithm of the user’s head orientation is used if the road does not have flat surfaces [8]. The system was validated by 10 participants to measure the controller accuracy [31].

Nasif and Khan [32] implemented a digital control using accelerometers to capture head movements. The sensors were placed on a cap to control five directions, and the data were sent by using radio frequency.

A multimodal interface control was developed by Fall and Latour [33] to be used for people with disabilities in upper limbs. The system is based on wireless sensor network. They developed a fusion algorithm for head movements using inertial sensors that were placed on earphones, and the communication system was realized using WiFi.

Marins et al. [34] used an Inertial Movement Unit (IMU) to capture user’s movements and operate the wheelchair. Data processing was carried out in Arduino and data classification using neural networks was developed in MATLAB^®^. The system was simulated in a closed environment with obstacles.

Prasad et al. [35] implemented a system with head movements in four directions. The system was controlled using an iOS application sending data to the motors.

Errico et al. [36] developed an interface in which a wheelchair is operated with head movements. The sensors were placed in a cap, and the movement directions were sent using radio frequency. The interface has an emergency button to make a call in the event of an accident. Other buttons of the system operate the wheelchair both indoors and outdoors.

Kader et al. [9] used 3-axis accelerometers to detect head movements. In addition, they implemented sonar sensors to detect obstacles in front or behind of the wheelchair, to avoid accidents. In case of emergency, the system sends a message using Global System for Mobile communications (GSM) to alert the family. The system has five directions, including Stop.

A control system was developed by Dey et al. [37]. The system has a seat belt to improve the safety of the user. Furthermore, it has ultrasonic sensors that detect obstacles. The wheelchair is powered by a solar panel, and uses sensors to be operated in case of improper head movements.

Manta et al. [26] realized a wheelchair command interface based on head movements. The system has two control modes, using simple commands and head movements. A 3D cartographic system is used to avoid obstacles. The control was based on an artificial vision system.

Other systems involve IMUs embedded into wearable devices, which have been used to athletes, in medical rehabilitation devices, among others. For this reason, some articles that have worked with IMUs in different areas are mentioned below.

Ayman et al. [38] recognized human activity by locating sensors on the hand, extracting multimodal data, in order to obtain healthier lifestyles. The activities carried out for the analysis were washing windows, cutting with a knife, eating, playing on a computer, and sending text messages using a keyboard and a pen. The fusion of gyroscope and magnetometer data allowed achieving an accuracy of 97.84% compared to other sensors. The combination of all the sensors in the system increased the accuracy to 98.87%. This accuracy indicates that sensor fusion was capable of improving daily human activity recognition rates.

Philpott et al. [39] developed an inexpensive and easy-to-use monitoring device within the sprint athletics community. Coaches monitor starting characteristics, allowing the coach to make technical adjustments. Twenty-five sprint start tests were done. The unit of measurement was accurate to 0.025 ± 0.024 s. Acceleration readings were higher, ranging between 1.15–2.60 m/s^2^, with a mean of 1.81 m/s^2^. An additional feature of this specific IMU allows the data to be provided to the user in real time, allowing the coach and the athlete to receive meaningful and useful in-training that can be implemented in their performance.

Setiawan et al. [40] designed a device to measure gait pattern using seven IMU (MPU6050 gyroscope and accelerometer). Communication is wireless. The recent interface design will improve heel strike, flat foot, heel lift off, and toe lift off. In this work, inertial sensors were used which provide Euler angles, analyzing gait and obtaining parameters for the rehabilitation of patients.

Gómez et al. [41] developed an IMU-based physical interface to measure foot attitude and a graphical interface that acts as a visual guide for patient rehabilitation. The data are displayed while the user performs dorsiflexion, flexion, eversion, and planar inversion movements. It can be used for therapy processes where the professional in charge refeeds the exercises based on the data collected. The best resolution ranges for data collection were ±2 gravities and ±250°/s. Rehabilitation could be achieved by reducing the variation in the angular position of the foot during therapy.

Gujarathi and Bhole [42] developed a gait analysis method to detect factors such as floor contact and non-contact. The experimental tests were based on walking a straight corridor with a distance of 40 m, and the data are processed in an algorithm to extract the period of events. The results identify the biomechanics of patients after surgeries. According to the authors, the sensor is effective for data acquisition and improves accuracy for gait analysis.

## 3. Materials and Method

The system configuration is described in this section. Section 3.1 describes the prototype electronic instrumentation and the transmission of data using the User Datagram Protocol (UDP). Section 3.2 describes the motion capture system configuration. The motion capture system IMOCAP-GIS was developed in the Software Research Group (GIS) at the Universidad Pedagógica y Tecnológica de Colombia (UPTC) [43]. Section 3.3 describes the transfer data method from the prototype to the computer.

### 3.1. Electronic Instrumentation of the Wheelchair Simulation Prototype

**Figure 1:**
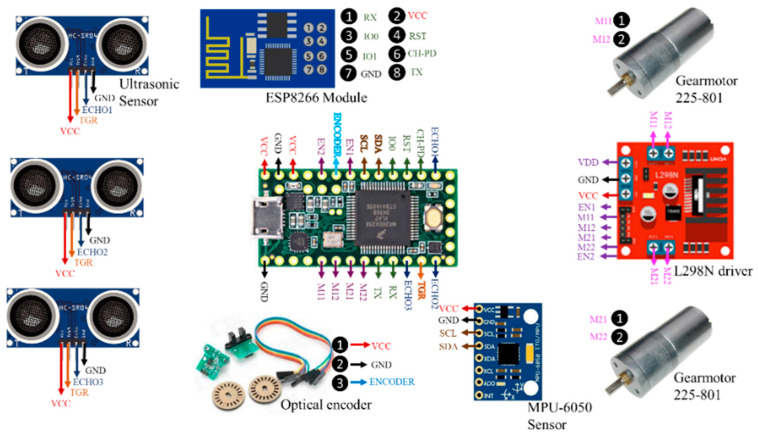
Devices connected to the microcontroller Teensy^®^ 3.2.

The wheelchair prototype instrumentation involves sensors, a control unit, actuators, a chassis, and communication protocols that were implemented for the correct performance of the system. The sensor distribution, control unit, communication modules, and actuators connected to the microcontroller are shown in Figure 1.

The prototype has a communication system through a WiFi module using UDP to operate the motors, considering their speed and direction, which are established through Pulse Width Modulation (PWM). Table 1 shows the command that receives the speed and direction to drive the prototype. The “@” command obtains sensors data of the prototype. PWM has a variation from 0 to 255; however, both motors only allow driving the prototype until a PWM of 100, obtained experimentally in function of the weight of the prototype.

**Figure 2:**
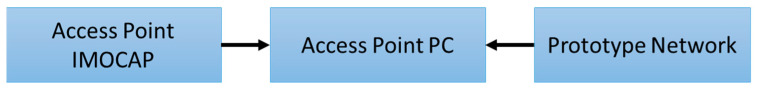
Block diagram among IMOCAP-GIS, prototype, and computer communicating via WiFi.

The system requires UDP communication protocol for the transmission and reception of data between sensors and microcontroller, and between the microcontroller and computer. The block diagram is shown in Figure 2.

### 3.2. IMOCAP-GIS Data Transfer Method

The IMOCAP-GIS platform consists of two Invensense MPU-9150 sensors, a master and a slave sensor, each with a 3-axis gyro with a sensitivity of 131 LSB/dps (degrees per second) and a range scale programmable from 250 to 2000 dps, a 3-axis accelerometer with a programmable range, a 3-axis magnetometer with a scale range up to 1200 mT, and a control unit with storage capacity and a computing device. The control unit can also serve as a temporary storage unit for offline data transmission, where the computing unit is not available [43]. A detailed analysis regarding the precision and definition of IMOCAP-GIS is described in [43].

The system was previously used in monitoring and rehabilitation processes, whose tests involved flexion and extension movements of a robotic arm. The system was configured in specific positions of the human body, as indicated [44], by means of a biomechanical model of the system involving three sections and three joints: The arm, joined to the shoulder and elbow joints; the forearm, attached to the elbow and wrist joints; and the section that belongs to the hand, attached to the forearm by the wrist joint. The Gaussian-Newtons, TRIAD, and Q methodology were used to estimate the orientation of a rigid body. TRIAD (Tri-Axis Attitude Determination) was used to obtain the orientation of the robot body by merging measurement vectors and reference vectors [44].

**Figure 3:**
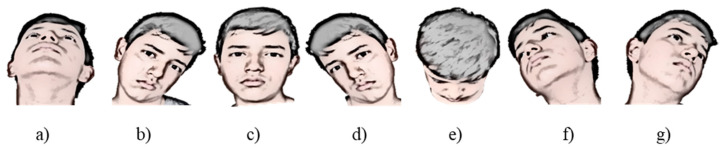
Head movements in seven directions to operate the prototype [45]: (**a**) Backward movement; (**b**) left movement; (**c**) stop; (**d**) right movement; (**e**) forward movement; (**f**) right-back movement; (**g**) left-back movement.

In this research, IMOCAP-GIS has the capability to configure wireless network or connect to one. In this case, the prototype configures the network, and IMOCAP-GIS connects to that network. When the network is configured to connect to the computer, it establishes a connection by having previously established the IP address and the local port. Then, the Euler angles (pitch, yaw, and roll) are generated from head movements. Figure 3 shows head movements for seven directions to operate the wheelchair prototype.

### 3.3. Data Transfer Method from Prototype to Computer

**Figure 4:**
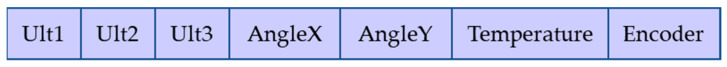
Vector structure of data reception that sends the prototype.

The prototype instrumentation processes information using a data vector separated by commas. This vector has data of three ultrasonics sensors, Euler angles of the inertial motion sensor (two angles are taken, since the third angle allows free mobility of the user without activating the prototype), temperature (parameter to know the status of the prototype), and optical encoder. The vector structure is shown in Figure 4. Data are processed by Python^TM^, a high-level programming language.

**Figure 5:**
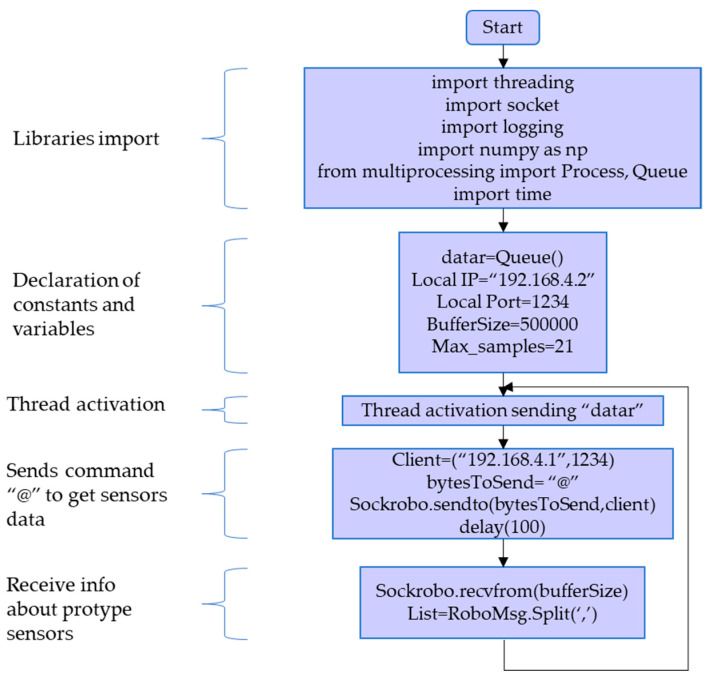
Flowchart of sending and receiving data between computer and prototype.

The flowchart for sending and receiving data between the computer and prototype is shown in Figure 5. Libraries importation allows to declare variables and constants, share data between threads, and start the communication to send information.

## 4. Results

This section is divided in three parts: First, the mathematical model based on physical characteristics of the prototype; second, the manual controller operated by graphical interface; and third, automatic controllers that uses fuzzy logic techniques.

### 4.1. Mathematical Model of the Prototype

**Figure 6:**
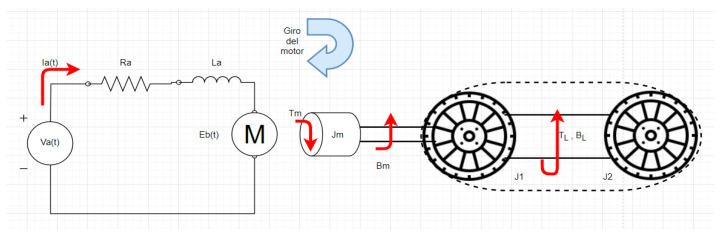
Graphic representation of the dynamic model of the wheelchair prototype.

The mathematical model developed here takes into account the electromechanical model of the DC motor connected to the load. In this case, the load is a closed rack pinion system. This system has its own characteristics related to the equivalent inertia present in two wheels, which are connected using track links and a static axle, as shown in Figure 6.

The model of the Figure 6 is described applying equations, where Equation (1) represents the voltage in the electrical circuit of the DC motors. Equation (2) relates the torque in the motor and the equivalent inertia of the track wheels. Equation (3) associates the armature current and torque in the motor. Finally, Equation (4) relates voltage motor to angular velocity motor.

(1) $$Va\left(t\right)=La\dot{i_{a}}\left(t\right)+Rai_{a}\left(t\right)+e_{b}\left(t\right),$$

(2) $$J_{m}\ddot{\theta_{m}}\left(t\right)+B_{m}\dot{\theta_{m}}\left(t\right)+B_{L}\dot{\theta_{m}}\left(t\right)+J_{e}\ddot{\theta_{m}}\left(t\right)=\tau_{m}\left(t\right),$$

(3) $$\tau_{m}\left(t\right)=K_{t}i_{a}\left(t\right),$$

(4) $$\dot{K_{e}\theta_{m}}\left(t\right)=e_{b}\left(t\right),$$

Equivalent inertia is represented as:

(5) $$J_{e}=J_{1}+J_{2}+mr_{1}^{2}\;\;,$$

Replacing in Equation (2), the equation is:

(6) $$J_{m}\ddot{\theta_{m}}\left(t\right)+B_{m}\dot{\theta_{m}}\left(t\right)+B_{L}\dot{\theta_{m}}\left(t\right)+\left(J_{1}+J_{2}+mr_{1}^{2}\right)\ddot{\theta_{m}}\left(t\right)=\tau_{m}\left(t\right)$$

The inertia of the wheel 1 is the same as the inertia of the wheel 2, and for that reason, it will be called “Jo”, representing two inertias. Therefore, the equations of the second order system are:

(7) $$\ddot{\theta_{m}}\left(t\right)=\frac{K_{t}i_{a}\left(t\right)}{J_{m}+2Jo+mr_{1}^{2}\;}+\frac{(B_{m}+B_{L})\dot{\theta_{m}}\left(t\right)}{J_{m}+2Jo+mr_{1}^{2}},$$

(8) $$\dot{i_{a}}\left(t\right)=\frac{V_{a}\left(t\right)}{La}-\frac{Ra}{La}i_{a}\left(t\right)-\frac{K_{e}}{La}\dot{\theta_{m}}\left(t\right),$$

The state space representation of the system is:

(9) $$\left[\begin{array}{c} \ddot{\theta_{m}}\left(t\right)\\ \dot{i_{a}}\left(t\right) \end{array}\right]=\left[\begin{array}{cc} -\frac{Ra}{La} & -\frac{K_{e}}{La}\\ \frac{K_{t}}{J_{m}+2Jo+mr_{1}^{2}\;} & \frac{\left(B_{m}+B_{L}\right)}{J_{m}+2Jo+mr_{1}^{2}} \end{array}\right]*\left[\begin{array}{c} i_{a}\left(t\right)\\ \dot{\theta_{m}}\left(t\right) \end{array}\right]+\left[\begin{array}{c} \frac{1}{La}\\ 0 \end{array}\right]*V_{a}\left(t\right),$$

The mathematical model theory developed in this research allows knowing the dynamic of the system in order to establish parameters of position, speed, and orientation of the fuzzy controllers, besides recognizing dynamic characteristics of the controllers to make comparisons between classic and intelligent control.

### 4.2. Manual Control

**Figure 7:**
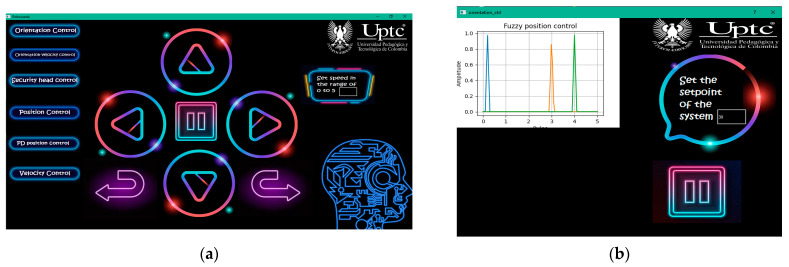
Graphical interface designed in Qt Creator: (**a**) Main window of the application; (**b**) new window displayed after clicking the automatic controller buttons.

Manual control was carried out considering the instrumentation of the system by sending commands (see Table 1) to drive the prototype. In addition, a graphical interface was designed to interact with the user through buttons and a speed setting. The main window is shown in Figure 7a, where the user can manually operate the system with buttons. Figure 7b shows the window that is generated when the user clicks an automatic controller button of the main window (left buttons).

### 4.3. Automatic Control

The automatic control of this research is based on intelligent control techniques. Then, the position, speed, and orientation control using fuzzy logic is developed.

#### 4.3.1. Fuzzy Position Control

**Figure 8:**
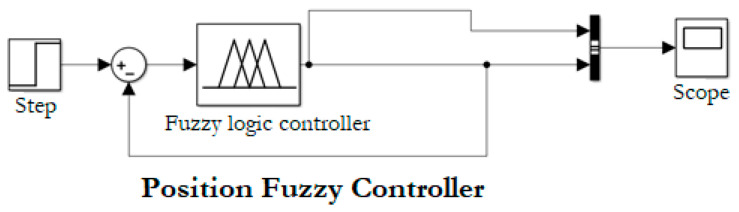
Block diagram of position fuzzy controller designed in “Simulink” of MATLAB^®^.

Position control establishes a reference, which is determined by the user depending on nearby obstacles located in front of the prototype. The block diagram of the fuzzy position controller is shown in Figure 8, in which there is a physical input called distance, and an output named system position.

**Figure 9:**
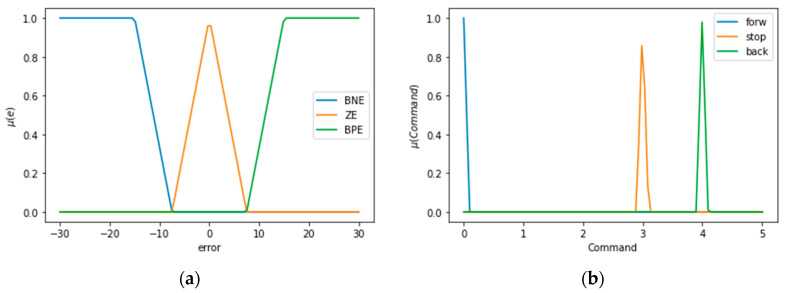
Linguistic variables for the error and controller output made in Google Colab: (**a**) linguistic variables of position error; (**b**) linguistic variables of output controller.

Linguistic variables allow establishing ranges in which the error will be in the desired range. The input variables are: “Big Negative Error” (BNE), when the difference between setpoint and measured value by the ultrasonic module is negative; “Big Positive Error” (BPE), when the difference is positive; and “Zero Error” (ZE), when the error equals to zero. The output variables to send commands to the prototype are “forward” (forw), “Stop” (stop), and “Come back” (back). In Figure 9a, the error established in the discourse universe is exposed. This value changes with the user input using the graphical interface. In Figure 9b, the output observed corresponds to speed commands sent to the prototype.

**Figure 10:**
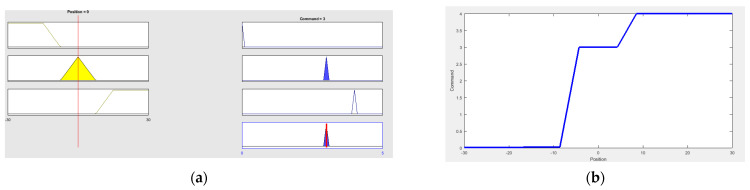
Position fuzzy control simulation using “FuzzyLogicDesigner” by MATLAB^®^: (**a**) Output fuzzy rules simulation; (**b**) Control surface.

Figure 10 shows the simulation of fuzzy rules and control surface using “FuzzyLogicDesigner” by MATLAB^®^. Table 2 shows the fuzzy rules table based on linguistic variables.

The fuzzification process is carried out employing membership functions in which the user enters a value, and the membership error is found in each fuzzy input set, obtaining the membership value. The defuzzification is implemented based on the centroid method, which takes the fuzzy output function and finds the central value of this function.

#### 4.3.2. Fuzzy Speed Control

Fuzzy speed control is executed utilizing a speed measure sent via optical encoder. The controller generates an output in revolutions per minute (rpm), which is measured within speed ranges stablished in Table 1. The ranges are related to speed commands and rpm on Table 3.

**Figure 11:**
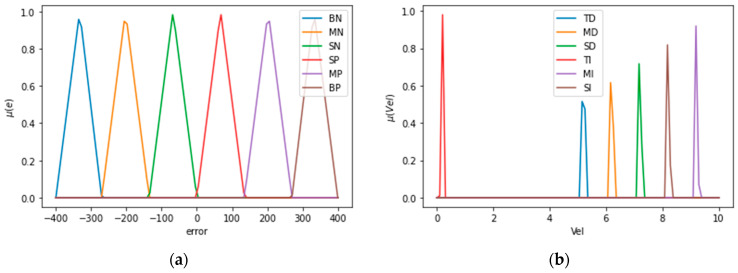
Linguistic variables for input error and output speed controller made in Google Colab: (**a**) Linguistic variables of speed input error; (**b**) linguistic variables of output speed controller.

Based on Table 3, the speed is parameterized for the fuzzy velocity control with respect to output controller command. The block diagram of the fuzzy speed controller has the same structure of the fuzzy position controller because both have one input and one output. The input error functions are shown in Figure 11a, which are: “Big Negative (BN), “Medium Negative” (MN), “Small Negative” (SN), “Small Positive” (SP), “Medium Positive” (MP), and “Big Positive” (BP). The output variables are observed in Figure 11b: “Small Increase” (SI), “Medium Increase” (MI), “Total Increase” (TI), “Small Decrease” (SD), “Medium Decrease” (MD), and “Total Decrease” (TD). The speed control simulated the cruising speed of cars.

Table 4 shows fuzzy rules. The response time of the controller was 335 ms.

#### 4.3.3. Fuzzy Orientation Controller with Constant Speed

**Figure 12:**
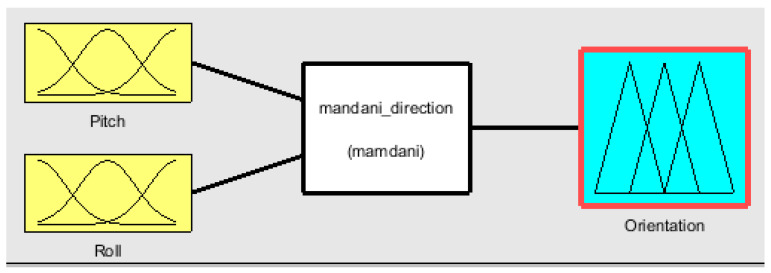
Block diagram for the fuzzy orientation controller.

Fuzzy orientation control uses IMOCAP-GIS system. The instrumentation of the motion capture system located in the cap allows sending Euler angles (pitch and roll), which are the input of the fuzzy controller. The block diagram of the controller is shown in Figure 12 from a MATLAB^®^ simulation.

**Figure 13:**
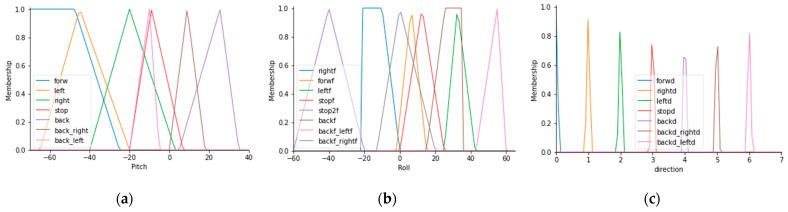
Membership functions for the inputs and outputs of the fuzzy orientation controller with constant speed: (**a**) Membership functions for pitch angle; (**b**) membership functions for roll angle; (**c**) membership functions for the output controller.

Figure 13a shows the membership functions for pitch angle. Figure 13b shows the membership functions for roll angle. Figure 13c describes the output membership functions, which is the prototype orientation.

**Figure 14:**
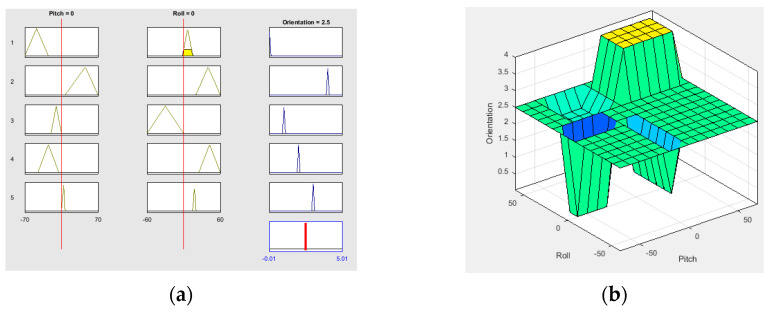
Fuzzy controller configuration employing “FuzzyLogicDesigner” by MATLAB^®^: (**a**) Fuzzy rules graphic; (**b**) control surface.

In this case, the conditionals use the structure: “IF …. AND…THEN…”. Figure 14 shows the fuzzy rules construction, inputs and outputs display, and the control orientation surface.

#### 4.3.4. Fuzzy Orientation Controller with Variable Speed

**Figure 15:**
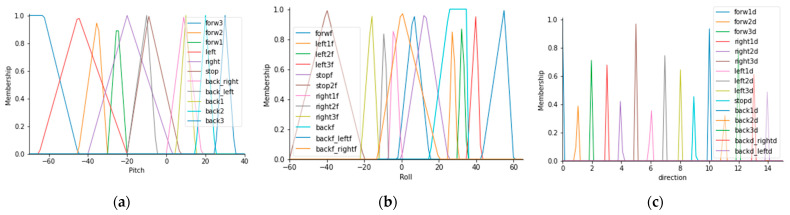
Membership functions for inputs and output of the orientation controller with variable speed: (**a**) Membership functions for the pitch angle; (**b**) membership functions for the roll angle; (**c**) membership functions for the output controller.

Fuzzy orientation controller with variable speed uses membership functions for three operating speeds taking into account head tilt angles. Figure 15a exhibits the functions for pitch angle. Figure 15b shows roll angle functions. Figure 15c shows the output controller where the system has three velocities for each direction: Forward, left, right, and back. The directions left-back and right-back run at full speed.

Table 5 shows the set of measurements of the tilt head angles for the orientation controller.

#### 4.3.5. Safety Control

**Figure 16:**
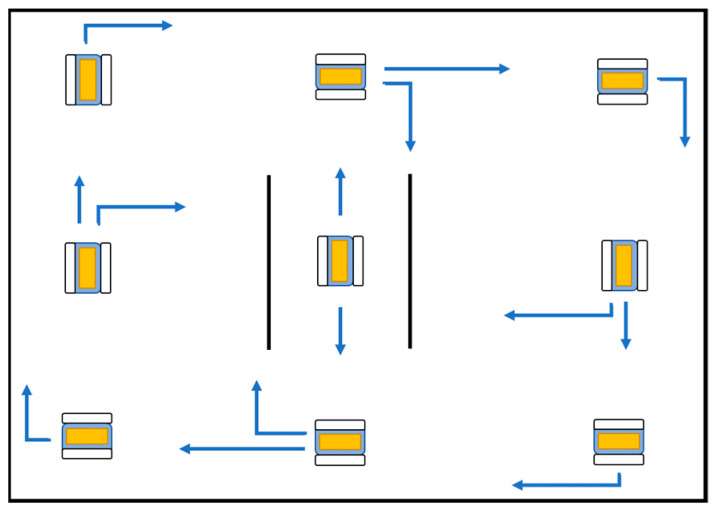
Room route with static obstacles.

The orientation control that was developed in the last section uses ultrasonic sensors to avoid nearby obstacles. The controller was implemented using conditionals in the code. Safety ranges are defined depending on the prototype location and considering the sensor measurements. Figure 16 shows a route inside a room with walls as obstacles. The algorithm is written when the prototype runs clockwise and counterclockwise. Two static obstacles were placed in the center of the route, representing areas in which the prototype can only be driven forward or backwards.

## 5. Discussion

The developed system has two aspects to be commented in this section. First, the performance of the electronic controllers based on system validation and compliance with design specifications. Second, user experience in terms of level of satisfaction with the system and recommendations.

### 5.1. Electronic System Response

The discussion in this section addresses details of the position, speed, and orientation controller designed here. The system has several elements in common with the research developed by Fall and Latour [33], such as communication systems through a WiFi module, whereas the graphical interface has similitudes with the works developed by Ruzaij et al. [8,29,30,31].

Marins et al. [34] developed data processing utilizing Arduino. In this case, the microcontroller was programmed using Teensy^®^, which is compatible with Arduino libraries. Emergency buttons were implemented in the system to disable controllers. On the other hand, distance sensors were used to ensure user safety, such as suggested by Prasad et al. [35].

The system used inertial sensors and distance sensors to be operated by the user of the prototype wheelchair; however, the ultrasonic range modules used in the system can be replaced by laser sensors to improve the accuracy of the measurements. On the other hand, the validation method of electronic controllers was analyzed using closed circuits that evaluate dynamic characteristics of the prototype. The inertial system was used due to its adaptability with the user, specifically users with disabilities in their lower and upper limbs.

It is worth commenting that although other systems used eye movements or brain signals to command a wheelchair, such as in [46,47], respectively, their response time is longer (about 2 s) compared to the system developed here (average of 100 ms). For instance, the route described by Gomes et al. [47] indicated a time lapse with an average of 1.56 s. In this research, the time used to make the route (shown in Table 7) had a variation between 22 and 62 s, with a distance of 10 m.

#### 5.1.1. Position Control

The stability of the systems is verified by the adequate operation of the controllers and satisfied design features, and the response time depends on parallelism used for sending and receiving data. The response time of the position controller was 225 ms, which was not possible to compare with results from the literature, as the authors of the literature reviewed did not quantitatively described the execution time of their controllers.

Njah and Jallouli [48] developed a fuzzy controller employing ultrasonic sensors with the aim of detecting obstacles. However, the response time of their system is unknown, and a specific environment is shown with randomly located obstacles.

Cui et al. [49] developed a fuzzy position system; however, their structure is different compared with this research, as their device operation is autonomous, meaning that it does not require user interaction at all, and involves angular variations when nearby obstacles are detected [49].

#### 5.1.2. Orientation Control

The response time of the orientation controllers is shown in Table 6. The fuzzy controller has a lower response time than the classic controller. The temporal response of the system was made using temporal measurement for each loop.

Saruchi et al. [50] developed a fuzzy orientation controller to drive their prototype, which has linguistic variables related to the input error and output error. In contrast, this research has Euler angles as inputs of the system and direction as output.

**Figure 17:**
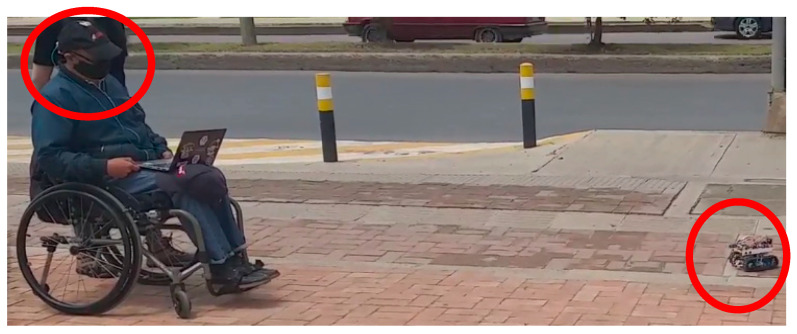
Validation test for the fuzzy variable speed orientation controller.

Yang et al. [51] designed a wheelchair control that also has Euler angles as inputs sent by a motion sensor, and their system outputs are directions of the wheelchair. However, their system has a test protocol to recognize motor intention of the user, calculating a recognition rate. In this research, the system is evaluated with user experience and response time of the controllers. The controller validation was carried out with a person with null mobility in lower limbs as shown in Figure 17.

### 5.2. User Satisfaction with the Developed System

**Figure 18:**
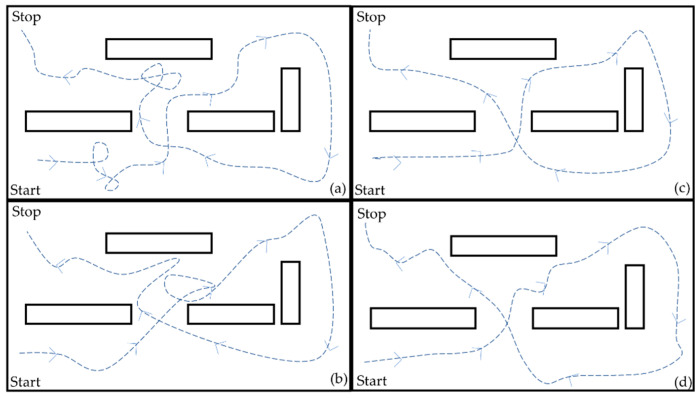
Routes carried out by User 1: (**a**) Route 1; (**b**) Route 2; (**c**) Route 3; (**d**) Route 4.

A route circuit was designed and carried out by 10 participants using the prototype with orientation controllers at constant and variable speed. The circuit was realized four times by each user to analyze the system performance, obstacle detection, and user experience. Figure 18 shows the four routes made by User 1. The first two routes were used for the system understanding and its interface. It was observed that in the latest tests, the user demonstrated a better control of the system executing movements.

Table 7 shows the execution time of the routes for the three controllers: Manual control, orientation controller with constant speed, and orientation controller with variable speed, obtaining different results in each case.

**Figure 19:**
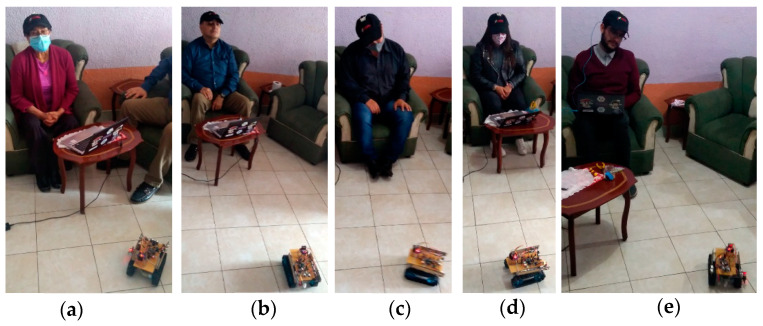
Participants utilizing orientation control: (**a**) Stop command (central head position); (**b**) backward command; (**c**) right command; (**d**) forward command; (**e**) left command.

Figure 19 shows the users manipulating the system with manual control and head movement function.

To measure the system usability, the literature describes different methodologies that analyzes the user experiences, such as the System Usability Scale (SUS) [43], that was used in this research. The SUS qualitative ranges are: From 0 to 25, worse; from 25 to 50, poor; between 50 and 70, good; from 70 to 80, very good; between 80 and 90, excellent; and greater than 90, unmatched. The users answered 10 questions, and each one was analyzed using an algorithm established for this measurement. The mean of the scores represents the SUS rate. The results of the survey are shown in Table 8. The statements are:

1. I think that I would like to use this feature frequently.
2. I found the feature unnecessarily complex.
3. I thought the feature was easy to use.
4. I think that I would need the support of a technical person to be able to use this feature.
5. I found the various functions in this feature were well integrated.
6. I thought there was too much inconsistency in this feature.
7. I would imagine that most people would learn to use this feature very quickly.
8. I found the feature very cumbersome to use.
9. I felt very confident using the feature.

**Table 8:** Application of System Usability Scale in participants.

User	Q1	Q2	Q3	Q4	Q5	Q6	Q7	Q8	Q9	Q10	Total
1	4	2	4	2	3	4	5	1	4	2	72.5
2	4	3	5	2	5	4	5	1	4	1	80
3	5	2	5	3	4	4	5	1	4	3	72.5
4	5	1	5	4	5	1	5	1	5	1	90
5	3	1	5	2	5	1	5	1	2	1	85
6	4	4	5	3	5	3	5	1	4	2	75
7	4	2	5	2	4	2	5	1	2	2	77.5
8	3	4	5	2	4	3	3	1	3	2	65
9	5	1	5	4	4	2	5	1	3	2	80
10	5	2	5	2	5	4	5	1	4	2	82.5

I needed to learn a lot of things before I could get going with this feature.

The results indicate that the SUS rates the system as “very good”, obtaining a mean score of 78. The system is accepted because the results are above 70. This qualitative rating was obtained from the analysis of the responses of all users, and taking into account the rating scale for the data provided by the questionnaire. In comparison, another study [52] implemented a SUS for rating a controller system of a wheelchair that incorporates a virtual hand to reach objects, and obtained a score of 65.3, which represents a qualitative rating of “good”. Despite having different instrumentation system and control, the scoring method is the same for both.

On the other hand, Montesano et al. [53] carried out an evaluation based on two perspectives: First, performance of the system and second, user experience. The system usability was evaluated quantitatively with four participants, unlike this research, conducted with 10 participants. Lastly, Onyango et al. [54] validated the prototype implementing an evaluation system that differs of the used in this research. The system had static obstacles and they evaluated the system safety when avoiding obstacles, such as in this research.

It is worth mentioning that in our experiments, the users referred to the system as “very good”, mentioning the importance of the manual controls to operate the prototype. The speed control option with constant speed allowed the users to drive long distances without manipulating the interface, making the prototype more autonomous. On the other hand, the orientation control developed here allowed the users to have control over the prototype without using their upper or lower limbs. The users mentioned that it is essential to have distance sensors activated all the time, as well as a safety control to avoid obstacles or accidents.

## 6. Conclusions

A position, speed, and orientation control system that implements head movements using a graphical interface was developed here. The position controller obtained a response time of 225 ms, and the speed control simulated the cruising speed of cars. The fuzzy orientation controllers responded in less time than on/off control, allowing the prototype to run in seven directions based on head movements. The participants had a high user experience, rating the system as “very good”, with a score of 78 out of 100 possible points. One test included the participation of a person with physical disability.

The users highlighted the use of distance sensors to detect nearby obstacles as a safety measure. However, the system cannot be used for long periods of time, as it can generate tiredness by the execution of several head movements. It is worth noting that our system can be used both by means of manual control (using the graphical interface) or processing head movements and tilt angles. The manual control was developed to be used by people with zero mobility in lower limbs whereas the processing of head movements is adequate to be used by people with zero mobility in lower and upper limbs.

The contribution of our system to the state of the art is based on the electronic development, executing the system in real time, and allowing setting parameters to be taken into account to validate it in a physical wheelchair.

## Figures and Tables

**Table 1:** Commands to operate the prototype motors via wireless connection.

Command	Velocity [PWM]	Orientation
@	-	-
0	255	Forward
1	255	Right
2	255	Left
3	0	Stop
4	255	Backward
5	100	Forward_vel1
6	140	Forward_vel2
7	180	Forward_vel3
8	210	Forward_vel4
9	240	Forward_vel5
a	255	Turn on its axis
b	100	Right_vel1
c	140	Right_vel2
d	180	Right_vel3
e	210	Right_vel4
f	240	Right_vel5
g	100	Left_vel1
h	140	Left_vel2
i	180	Left_vel3
j	210	Left_vel4
k	240	Left_vel5
l	100	Backward_vel1
m	140	Backward_vel2
n	180	Backward_vel3
o	210	Backward_vel4
p	240	Backward_vel5
q	255	Backward_right
r	255	Backward_left

**Table 2:** Fuzzy rules based on linguistic variables for position controller.

**Error**	BNE	ZE	BPE
**Output**	forward	stop	back

**Table 3:** Speed ranges in RPM and speed commands relationship.

Velocity Level	Command	Velocity [rpm]
0	5	300–318
1	6	300–320
2	7	315–345
3	8	345–355
4	9	350–360
5	0	355–365

**Table 4:** Fuzzy rules based on linguistic variables for speed controller.

Velocity Error	BN	MN	SN	SP	MP	BP
**Velocity**	TD	MD	SD	SI	MI	TI

**Table 5:** Roll and Pitch angles for head orientation.

Orientation	Roll Angle	Pitch Angle
Forward	(−70, −25)	(−2, 15)
Left	(−65, −25)	(25, 42.5)
Right	(−40, 3)	(−22, 0)
Stop	(−20, 7)	(0, 25)
Backward	(5, 35)	(15, 35)
Back-left	(0, 18)	(−13, 20)
Back-right	(−20, −5)	(43, 60)

**Table 6:** Response time of orientation controllers designed.

Type Controller	Response Time [ms]
Fuzzy	25
On/Off	30

**Table 7:** Response time of controllers in each participant.

Participant	Manual Control	Orientation Control	Orientation Velocity Control
1	40	27	32
2	38	29	34
3	36	24	32
4	45	60	90
5	22	31	38
6	44	34	52
7	22	27	24
8	45	40	42
9	34	35	36
10	52	58	62

## Data Availability

Informed consent of the participants are available writing to: aura.gonzalez@uptc.edu.co.
